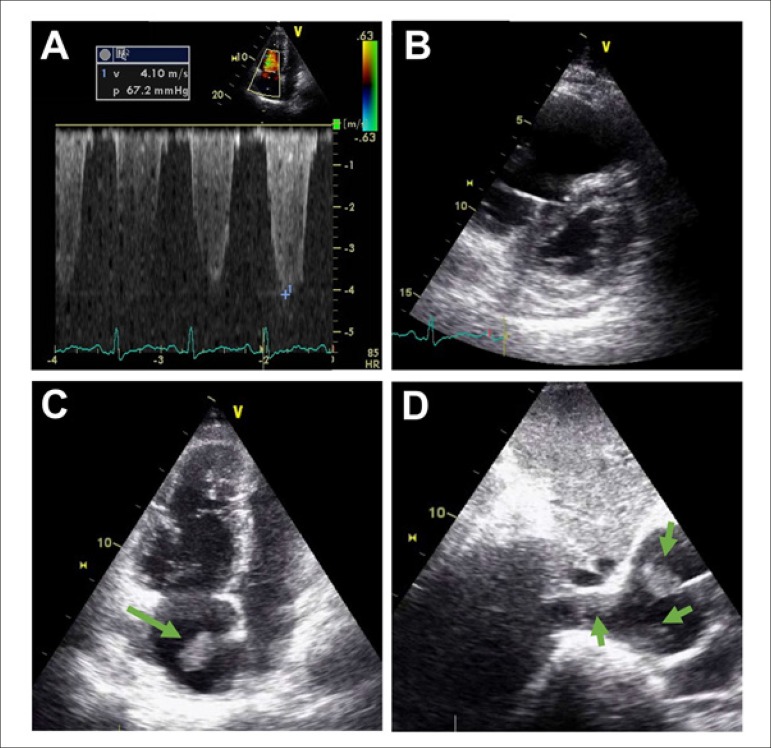# Multiple Thrombi in the Inferior Vena Cava and Right Atrium – Recurrent Thromboembolism Due to Polycythemia Vera

**DOI:** 10.5935/abc.20160088

**Published:** 2016-08

**Authors:** Kevin Domingues, Liliana Marta, Marisa Peres, Isabel Monteiro, Margarida Leal

**Affiliations:** Hospital de Santarém – Portugal

**Keywords:** Polycythemia Vera, Pulmonary Embolism/mortality, Thrombosis, Vena Cava, Inferior, Heart Atria, Tomography, X Ray Computed, Echocardiography

Thromboembolic events are a major cause of morbidity in patients with polycythemia vera (PV), accounting for a third of the deaths in this population. PV is a clonal disorder in which disturbed hematopoiesis leads to increased erythropoiesis, myelopoiesis, and/or megakaryopoiesis, characterizing this disorder as a prothrombotic state.

We report here the case of a 72-year-old man with a history of hypertension, type 2 diabetes mellitus, and implantation of a DDDR pacemaker 6 years before due to sick sinus syndrome. He also had an unclear, recent history of high hemoglobin levels and leukocytosis. He had been admitted three times in less than 1 year for acute pulmonary thromboembolism (PTE), despite being on anticoagulation therapy (one time on rivaroxaban, another on warfarin with adequate INR). A computed tomography scan revealed occlusive bilateral thrombi in the lower lobe arteries. Echocardiography was performed and showed enlargement of the right cavities, D-shaped left ventricle, severe pulmonary hypertension, and right ventricular dysfunction (tissue Doppler imaging = 8 cm/s). Multiple thrombi were present in the right atrium (some attached to the pacemaker lead) and in the inferior vena cava. Nevertheless, the patient was hemodynamically stable and comfortable with an oxygen mask.

Therapeutic options were discussed, and a conservative approach with anticoagulation was adopted. A genetic testing was performed, revealing a *JAK2-V617F* gene mutation, which along with a hemoglobin level > 18.5 g/dL confirmed the diagnosis of PV according to 2008 World Health Organization diagnostic criteria. The patient was started on cytoreductive therapy with hydroxyurea and phlebotomies but presented again with another PTE 1 month later. We then decided to increase the target INR to 3–4, and until this date, no new thrombotic events have been registered.

## Figures and Tables

**Figure:**